# Economics of Philanthropy

**Published:** 1898-03-12

**Authors:** 


### The Institutional Workshop.

### ECONOMICS OF PHILANTHROPY.

### IV.—PEASANT PROPRIETORSHIP.

The moral improvement worked by the institution of long leases suggests what might be the good effect of peasant proprietorship. In almost all Continental countries a great part of the land is divided into small estates, or if they are really farms, that is, if they are rented from a large landowner, the rent is paid in produce, and not in money. This latter system—the métayer system, as it is called, from the fact that the usual proportion which the landlord takes is the half —makes the proprietor share equally with the tenant the profit of good years, and the losses of bad ones. There are no rebates of rent, because the rent is always a fixed proportion of the produce, and the landlord has a more direct and conscious interest in keeping the estate in good order than if he received a money rent. As custom forbids the eviction of a tenant, the latter has almost the feeling of proprietorship; and the security of his tenure encourages him to spend both labour and capital on the ground, while the fact that the landlord shares the profit induces the latter to lend money for the stocking and development of the farm. The métayer is thus more akin to the peasant proprietor than to a tenant at will; and anything we have to say of the effect of such proprietorship applies, though in a modified degree, to his tenure.

All observers, though many of them are prejudiced in favour of all things English, admit the industry, prudence, and frugality of the peasants of the Continental countries. The French peasant may stand for a type of all; for the same qualities are found everywhere. Their main characteristic is a tireless industry. Professor Walker says, "the true savings bank is the land"; and this is so because it is always there, ready to take an extra hour's labour, an extra shilling expended in tools, or manure, or seeds; and willing to give a good return for what it gets. Travellers who take any note of the little farms in the countries they have visited unite in expressing their surprise at the number and richness of the crops that are grown on soils which are not naturally richer than those which English farmers, employing hired labour, cannot work profitably. The reason lies simply in this matter of paying for labour. "A labour of love" is proverbially more heartily done than one undertaken for hire; but, apart from this, it must be remembered that even if the hired worker was willing to work as hard for his master as the peasant proprietor does for himself, his necessary wages would consume so much of the produce of his work that the master would have only a very insignificant profit; and, if added to rent, perhaps none at all. The man who is cultivating his own soil does not reckon his time as money. The land keeps him and his family, which is the one thing he asks of it, and what it yields beyond the necessaries of life is in his eyes pure profit. He no more sets to work to reckon up how many hours he has worked in the year, and at what rate of pay, than a mother does in thinking of the time she spends in attending to her children. The good results of such cultivation are gained by labour of the most unwearying kind; but the labour is made sweet by the feeling that all the gains belong to the worker. As Arthur Young said a century ago, when studying the condition of the peasantry of France, "Give a man the secure possession of a bleak rock, and he will turn it into a garden; give him a nine years' lease of a garden, and he will convert it into a desert" ("Travels in France," vol. 1, p. 88).

Another characteristic of these peasant farmers is their frugality. English observers have sometimes taken this as a proof that this mode of cultivation did nothing to add more to the comfort of the workers than did the paid wages of the English agricultural labourer. But if the peasant lives poorly, it is because he is economical, perhaps penurious, rather than because he is poor. He may eat black bread, and rarely taste meat; but it is not because he cannot afford better, but because he prefers to save money. At the same time, it must be remembered that the prosperity of the peasant proprietor, which those who wish to see the system introduced more largely into this country dilate on, is won and maintained only by the most unwearying industry and the closest economy. That such a race of men are a source of strength to any country is undeniable. The qualities which make them what they are have a moral value that cannot be over-estimated, and for this reason it would be well if it were made easy for agricultural labourers to acquire suitable plots of ground in absolute ownership; but it would be a mistake to flatter them by hopes of leading an easier life than they do now. The great advantage would undoubtedly be the increase of production, and the development of habits of greater industry and thrift.

We have not spoken of another characteristic of the peasant proprietor, his determination to limit the number of his family; because we think that this is a tendency which, in an empire like ours, is not to be encouraged. Our own colonies are not yet fully peopled, and, besides these, the United States and the Transvaal claim every year a large number of our sons. To meet this drain upon our population we need larger families than a French peasant would care to rear; and it seems certain that the ownership of land, in all ranks, tends to make the handing down of the family estate intact an object to be aimed at. With large estates this can be done by entails, and the younger members of the family may be provided for by charges on the estate; but this plan is out of the question with small properties. The alternatives there are the cutting up of the ground into small inheritances, none of which would singly support a family (and in this case the owners would soon fall into a pitiable state of poverty) or the severest limitation of the family, which, in a nation with such outlets for population as ours, is by no means desirable. It is probable, however, that any pronounced revival of agricultural prosperity must take the form of peasant proprietorship rather than in new methods of working large farms held at a rental and worked by hired labour. This cannot compete, on the one hand, with the tireless energy of the labourer of the Continent, nor on the other with the virgin soils of America. The double expense of rent and labour would leave too small a profit to tempt the farmer who wants to live rather like his landlord than like his labourers. But, with land going out of cultivation which is as fertile as that out of which a French, Belgian, Dutch, or Swiss peasant farmer could make a living and save money, it seems a pity that the experiment of creating small properties with peasant owners could not be tried. We cannot conclude this subject better than by giving Professor Marshall's conclusions on the question:—

"Though peasant proprietorship, as a system, is unsuited to the economic conditions of England, to her soil, her climate, and the temper of her people, yet there are a few peasant proprietors in England who are perfectly happy in this condition; and there are a few others who would buy small plots of land, and would live happily on them, if they could get just what they wanted where they wanted it. Their temper is such that they do not mind working hard and living sparely, provided they need call no one master; they love quiet, and dislike excitement; and they have a great capacity for growing fond of land. Reasonable opportunity should be given to such people to invest their savings in small plots of land, on which they may raise suitable crops with their own hands; and, at the very least, the present grievous legal charges on the transfer of small plots should be diminished" ("Economics of Industry," Vol. I., page 740).

Up to this point we have been speaking of holdings which are large enough to maintain a whole family in decent comfort. But the majority of English allotments, whether the property of the cultivators or rented, are too small for this. In 1890 there were 455,005 allotments of less than one acre, while other small holdings up to 50 acres numbered 409,422. On the Continent an average holding for a peasant proprietor is about six acres. It is thus seen that the majority of small holdings in England are not enough to form the whole maintenance of a family, while some at least of the others must be cultivated by hired labour. Of the latter we shall not speak, as it is the cost of labour that so often eats up the profits of farming; though perhaps in these small farms, where the employer and the labourer work side by side, this is not so frequently the case as in larger ones. The others supply work which labourers may attend to in their leisure time. Mill objects to them on this ground, fearing that such an aid to the ordinary earnings will have the effect of reducing wages, as the old allowance system did. That this is the usual effect of "by-work" of any kind is admitted; and, especially in women's employments, its influence in lowering wages is very perceptible. But, in this matter of agriculture, the evil effects, if such there are, seem to be out-weighed by the good. In our present condition with so many of our young men either going to towns or emigrating, induced by the prospect of better wages and the hope of advancement in life, it seems that anything that makes agricultural labour more attractive is a good thing. It means a great deal to Britain if it can keep within its borders a race of men, strong, healthy, self-supporting, able to fight its battles, if need be. If, by any means, the country is enabled to support a greater number of men than it was formerly, the country is so much the richer; and, so far, we have perceived no tendency, through the increase of allotments, towards the diminution of the wages of agricultural labourers. And it must be admitted that in the event of a war with any such combination of naval powers as would be strong enough to blockade our ports, when our insular position might be a source of weakness instead of strength to us, it would become a matter of importance how many men we could rear and feed. Anything that develops our agriculture is useful. In this era, people are not so likely as they were a century ago to remain in one place, if that place offers them less in the way of remuneration, liberty, and expectation of better things than others; the press and the post have changed all that, and we are willing to hope that an extension of allotments may really put our agricultural population in a position of such comfort as to justify their being contented in it.

